# Editorial: Advances in musculoskeletal imaging

**DOI:** 10.3389/fphys.2024.1535622

**Published:** 2024-12-06

**Authors:** Victor Casula, Simo Saarakkala, Jukka Hirvasniemi

**Affiliations:** ^1^ Research Unit of Health Sciences and Technology, Faculty of Medicine, University of Oulu, Oulu, Finland; ^2^ Department of Diagnostic Radiology, Oulu University Hospital, Oulu, Finland; ^3^ Department of Radiology and Nuclear Medicine, Erasmus MC University Medical Center Rotterdam, Rotterdam, Netherlands; ^4^ Department of Biomechanical Engineering, Delft University of Technology, Delft, Netherlands

**Keywords:** medical image analysis, musculoskelatal disorders, magnetic resonace imaging (MRI), artificial intelligence-AI, spine, muscle, spondyloarthritis

## Introduction

Musculoskeletal disorders such as osteoarthritis, bone fractures, osteoporosis, or ligament injuries are highly prevalent conditions affecting muscles, bones, joints, and adjacent connective tissues. In 2020, they affected over 1.63 billion people worldwide (Gill et al., 2023). Musculoskeletal disorders cause various symptoms such as pain and mobility limitations, and they have a tremendous impact on the quality of life of an individual imposing a large economic burden to a society (Liu et al., 2022; Parto et al., 2023). The underlying pathophysiological processes and clinical manifestations vary depending on the affected tissue and specific disorder.

Imaging provides visualization of the anatomy and physiological processes of the body and plays an important role in the diagnostics of musculoskeletal disorders both in clinical practice and research. Conventional radiography and computed tomography (CT) are usually used to assess changes in the mineralized tissues, such as subchondral bones, whereas magnetic resonance imaging (MRI) is best suited for assessing changes in the soft tissues, for example, damage in tendons, ligaments, menisci, and cartilage. Novel imaging, image processing, and image analysis techniques have a great potential to enable earlier diagnosis and deeper understanding of musculoskeletal disorders and pathophysiological processes related to them (Kijowski and Fritz, 2023). Furthermore, artificial intelligence (AI) techniques have a great potential to enhance each component in the imaging chain (Burns et al., 2020; Debs and Fayad, 2023; Fritz et al., 2022; Gitto et al., 2024; Hirschmann et al., 2019).

The aim of this Research Topic was to collect articles showcasing applications of novel and innovative imaging, image processing, and image analysis techniques for assessing musculoskeletal disorders and the underlying pathophysiological processes. Although musculoskeletal conditions can affect various joints, and no specific restrictions were placed on this Research Topic, the articles included in this Research Topic focused on disorders involving the spine, spinal joints, and associated muscles.

Two articles studied patients with axial spondyloarthritis, which is a chronic inflammatory disease primarily affecting the spine and sacroiliac joints (Sieper and Poddubnyy, 2017). MRI is an important tool in diagnostics of spondyloarthritis since it can reveal active inflammation in the sacroiliac joints and spine before detectable structural changes on plain radiographs (Mauro et al., 2024).

Lee et al. presented an AI method for the detection of sacroiliitis on MRI in patients with axial spondyloarthritis. They used Faster R-CNN with ResNet-50 to extract sacroiliac joint regions from the MRI and VGG-19 to classify if the joint had sacroiliitis according to the Assessment in SpondyloArthritis international Society (ASAS) definition (Sieper et al., 2009). The best performing model was trained using data augmentation and maximum intensity projection and had area under the receiving operating characteristics curve values above 0.80 at the slice level and participant level classification tasks.

In a retrospective clinical study, Yang et al. explored the potential of MRI relaxometry and mucosal-associated invariant T cells (MAIT) related parameters in assessing inflammatory activity in patients with axial spondyloarthritis. The authors found that prolonged T1 relaxation times, lower percentages of total MAIT cells, and higher percentages of CD69+MAIT cells were associated with higher disease activity. Furthermore, combining T1 relaxation times with percentages of CD69+MAIT cells demonstrated greater accuracy in distinguishing between active and inactive disease states compared to single parameters. These findings suggest that T1 relaxation time values, percentages of MAIT cells, and CD69+MAIT cells are indicators of inflammatory activity in axial spondyloarthritis, and the combined use of T1 mapping and MAIT cell activation levels may provide a more comprehensive assessment of disease severity.

Athertya et al. proposed to use a strong T1 weighting in 3D fat-suppressed spoiled gradient recalled-echo MRI sequence to enhance visualization of the cartilaginous endplate. Conventional clinical imaging often lacks sufficient contrast, making detailed visualization of the cartilaginous endplate challenging. The proposed MRI sequence demonstrated superior contrast between the cartilaginous endplate and surrounding tissues while enabling detection of abnormalities in cartilaginous endplate morphology. The sequence offers fast acquisition and is commercially available on clinical scanners, making it easily translatable to clinical settings, and holds potential for improved spinal diagnosis, treatment planning, and longitudinal studies of therapeutic effects.

Maggioni et al. used a multiparametric MRI measurement protocol to extract quantitative values of (water) T2, fat fraction, T1, and intra voxel incoherent motion (IVIM) diffusion parameters to assess training-associated changes of the lumbar back muscle. They reported differences in muscle water T2, fat fraction, and pseudo-diffusion coefficient linked to microcirculatory blood flow in muscle tissue between the trained and untrained cohorts. Furthermore, diffusion coefficients were reported to provide additional differentiation between the trained and untrained cohorts. They concluded that quantitative MR parameters have potential to detect and quantify long-term effects of training.

In conclusion, this Research Topic highlighted studies using imaging for the assessment of musculoskeletal disorders, with a particular focus on the back. Overall, AI methods and advanced imaging techniques have potential to improve diagnostic accuracy and deepen our understanding of musculoskeletal disorders.
